# The Rate of Appendicular Neoplasm in Patients Who Underwent Appendectomy for Acute Appendicitis at King Abdulaziz Medical City, Riyadh

**DOI:** 10.7759/cureus.31581

**Published:** 2022-11-16

**Authors:** Mohammad Y Alamoudi, Nasser M Alkahtani, Yahea M Aldosari, Sarah Marie, Abdullah Abdulrahman Ashmawi, Yazeed J Alshaalan, Faisal K Alabdulrahman, Zeyad Yousef, Mohammed F Alserhani

**Affiliations:** 1 Medical Intern, King Saud Bin Abdulaziz University for Health Sciences College of Medicine, Riyadh, SAU; 2 Surgery, King Abdulaziz Medical City Riyadh, Riyadh, SAU; 3 Internal Medicine, King Saud Bin Abdulaziz University for Health Sciences College of Medicine, Riyadh, SAU

**Keywords:** tertiary medical hospital in riyadh, alvarado score, appendiceal carcinoid, acute appendicitis, appendiceal mass

## Abstract

Background

Appendicitis is a common reason for hospitalization. Rarely do people with acute appendicitis have an appendiceal mass called an abscesses or inflamed phlegmon. The goal of this study was to determine the prevalence of different appendiceal tumors including neuroendocrine tumors, adenocarcinoma, carcinoid, and mucinous and evaluate patient demographic data (e.g., age and gender) at a major tertiary care center in Riyadh, Saudi Arabia.

Materials and methods

We conducted a cross-sectional analytical review of patient records of 1513 patients who received an appendectomy and were diagnosed with acute appendicitis from 2015 to 2020 at King Abdulaziz Medical City, Riyadh, Saudi Arabia. We used nonprobability sampling to collect the sample. The study included patients older than 14 years. We also recorded patient demographic information, including age, gender, history, and final pathology.

Results

The mean age of our study population was 27.9 years (standard deviation [SD], 12.3 years). Our study had 958 male patients and 555 female patients. One thousand four hundred fifty-eight patients (96.3%) had right lower quadrant (RLQ) tenderness, and 228 patients had fever (15.0%). One thousand one hundred thirteen patients (73.5%) had rebound tenderness, 1,178 had nausea (77.8%), and 1,100 had high white blood cell (WBC) counts (72.7%). One thousand four hundred eighty-six patients received laparoscopic surgery (98.2%). Most patients (95.3%; n=1,443) had no postoperative complications. Appendicitis pathology was present in 1,381 patients (91.3%). Only 15 patients (1%) had tumor-related pathology, and these patients were significantly older than patients with nontumor-related pathology (p<.001) and had less RLQ pain, rebound tenderness, and pain migration but higher WBC counts. Pain migration was significantly inversely correlated with age: as age increased, pain migration was reported less often (odds ratio, 0.99, 95% confidence interval, 0.98 to 0.99; p=0.001).

Conclusion

This study aimed to determine the prevalence and types of appendiceal tumors in cases of acute appendicitis and the corresponding patient demographic data at a major tertiary care center in Riyadh, Saudi Arabia. According to our results, patients with appendicitis present with fever, rebound tenderness, nausea, and high WBC count. Appendiceal masses mainly occur in a later age group with less migration of pain and high WBC count. However, migration of pain is inversely related to age. Physicians treating patients with acute appendicitis should bear these data in mind and consider the presence of appendiceal tumors in appropriate patients.

## Introduction

Appendicitis causes over 300,000 hospitalizations annually alone in the US. The most frequent gastrointestinal illness, appendicitis, results in more than 40,000 hospitalizations in England each year [1]. The most frequent reason for an acute abdomen needing surgery is acute appendicitis. Acute appendicitis is chiefly diagnosed clinically, so many individuals have a typical medical history and physical examination results. Most patients describe peri-umbilical colicky pain as their history. This pain worsens over the course of the first 24 hours, turning into a constant, sharp pain, and migrating to the right iliac fossa. The most reliable clinical findings of acute appendicitis are percussion tenderness, guarding, and rebound tenderness [2].

Appendiceal masses occur in approximately 2% to 6% of cases of acute appendicitis; these are often called abscesses or inflamed phlegmon [3]. Appendiceal malignancies make up around 1% of large intestine cancers. In more than 50% of instances, acute appendicitis is the first sign of primary appendicular malignancies, which surprises the therapist and patient [4]. According to reports, inflamed appendiceal masses occur in 2% to 6% of acute appendicitis patients [5], and newer studies show an increased prevalence of cancerous tumors between 5.9% and 12% [6,7].

There is a paucity of data on appendiceal tumors presenting as acute appendicitis in the literature locally. Therefore, the goal of this study was to determine the prevalence of different appendiceal tumors including neuroendocrine tumors, adenocarcinoma, carcinoid, and mucinous and highlight patient demographics and tumor disease characteristics at a major tertiary care center in Riyadh, Saudi Arabia. 

## Materials and methods

We conducted a retrospective, cross-sectional analytical review of patient records of patients who received an appendectomy at King Abdulaziz Medical City, a major tertiary care center in Riyadh, Saudi Arabia, from 2015 to 2020. The study included patients older than 14 who underwent laparoscopic or open appendectomy who were diagnosed preoperatively with acute appendicitis either clinically by Alvarado score of a minimum of 4, or by imaging regardless of Alvarado score. The study excluded any patient who had an incidental finding of appendicitis through imaging for other medical reasons or underwent appendectomy through an incidental finding for other procedures in the operating room. The Institutional Review Board of King Abdullah International Medical Research Center provided ethical approval of the study (Ref. No. RYD-21-419812-152004).

We used nonprobability sampling to collect study data from patient medical records in the National Guard Health Affairs, Riyadh, Saudi Arabia, via the BESTCare System (ezCareTech, Seoul, South Korea). Patient demographics, including age, gender, and final pathology were collected. We assessed the number of patients with appendicitis having migration of pain to the right lower quadrant, anorexia, and nausea/vomiting. We also recorded tenderness in the right lower quadrant, rebound tenderness, and a body temperature higher than 37.3 °C. Laboratory findings such as leukocytosis and neutrophilia were collected, and we compared laparoscopic and open appendectomies and postoperative concerns against those from other diagnoses.

Our study used the Alvarado score, a 10-point scoring system that uses eight parameters to estimate the likelihood of appendicitis with parameters related to subjective findings such as migration of pain to the right lower quadrant (1 point), anorexia (1 point), and nausea/vomiting (1 point). It also measures objective findings such as tenderness in the right lower quadrant (2 points), rebound tenderness (1 point), and a temperature higher than 37.3 °C (1 point). Laboratory findings such as leukocytosis (2 points) and neutrophilia (1 point) are also scored [8].

The higher the Alvarado score, the higher probability of appendicitis. An Alvarado score of less than 4 can rule out appendicitis, and a score of 5 to 6 indicates a moderate probability of appendicitis and requires further investigation. A score of 7 or more indicates a high probability for appendicitis, and a patient can be managed accordingly [8]. The Alvarado score was included in this study to assess which parameters are related to the presence of tumors. 

The pathology results were collected via the BestCare system and different types of masses were subcategorized into normal, acute appendicitis, neuroendocrine tumor, adenocarcinoma, carcinoid, mucinous, and other benign histopathology such as tubulovillous adenoma, benign myxoid mesenchymal lesions and endometriosis in the appendix.

Statistical analysis 

Patients' basic descriptive data and clinical presentation, and complications were summarized as frequencies and percentages. Continuous data of the descriptive statistics were presented as mean and standard deviation (SD). Pearson Chi-square test was used to analyze categorical variables, and Student's T-test was used for continuous variables. Logistic regression of age and pain migration were used to predict the probability of pain migration with age. Statistical analysis was performed with STATA version 15.0 (StataCorp, College Station, TX, USA) with statistical significance defined at p < 0.05. 

## Results

Our cohort of appendectomy patients consisted of 1513 patients (958 male patients, 555 female patients) with a mean age of 27.9 years (SD, 12.3 years). Table 1 represents the demographics, symptoms, and laboratory findings. Right lower quadrant (RLQ) tenderness was the most common symptom (n=1,458; 96.3%), followed by rebound tenderness (n=1,113; 73.6%), nausea (n=1,178; 77.9%), and high white blood cell (WBC) counts (n=1,100; 72.7%). Most appendectomies were performed laparoscopically (n=1,486, 98.2%)

**Table 1:** Patient demographic data, symptoms, laboratory values, and procedure data Abbreviations: RLQ, right lower quadrant; WBC, white blood cells.

Variable	N	Percentage (%)
Male Patients	958	63%
Female Patients	555	37%
RLQ Tenderness	1,458	96.4%
Fever (>37.3 °C)	228	15.1%
Rebound Tenderness	1,113	73.6%
Anorexia	858	56.7%
Nausea and Vomiting	1,178	77.9%
Migration of Pain to RLQ	865	57.2%
High WBC >10,000 cells/µL	1,100	72.7%
Neutrophils >75%	897	59.3%
Laparoscopic Procedure	1,486	98.2%
Prognosis concern	70	4.6%

Table 2 represents postoperative complication data. While 1,443 patients (95.4%) had no complications, 70 patients (4.6%) had complications. The most common complications were peritoneal collections (n=21; 1.4%), wound infection/pus (n=19; 1.3%), fever (n=3; 0.2%), and ileus (n=3; 0.2%).

**Table 2:** Postoperative complication data *Other conditions such as minimal bloody discharge, bleeding, small bowel obstruction, cecal diverticulitis, vomiting, dysuria, shortness of breath, constipation, thrombosis, epilepsy, diarrhea.

Complication Type	N	Percentage (%)
None	1,443	95.4%
Infection	1	0.07%
Peritoneal collections	21	1.4%
Fever	3	0.2%
Ileus	3	0.2%
Wound infection, pus	19	1.3%
Other*	23	1.5%

Table 3 represents the Alvarado scores in our patient population. An Alvarado score of 8 occurred in 345 patients (22.8%), followed by Alvarado scores of 7 in 269 patients (17.78%) and 9 in 239 individuals (15.8%), respectively. 

**Table 3:** Alvarado scores

Alvarado Score	N	Percentage (%)
0	1	0.07%
1	7	0.46%
2	28	1.85%
3	60	3.97%
4	107	7.07%
5	183	12.1%
6	216	14.28%
7	269	17.78%
8	345	22.8%
9	239	15.8%
10	58	3.83%

The nature of the histopathology discovered during appendectomy is represented in Table 4. Most patients had typical acute appendicitis (n=1,381; 91.3%), while 109 had a healthy appendix (7.2%). Tumors were rare in our study, occurring in only 15 patients (1%), 10 of whom had mucinous appendiceal neoplasms (0.7%). Table 5 compares the pathology data against patient demographic data. Patients with tumors were significantly older (mean age, 41 years; p<.001) than those without tumors (mean age, 27 years; p<.001), and they had less RLQ, rebound tenderness, and migration of pain. Patients with tumors had higher WBC counts than those without. 

**Table 4:** Pathologies of tumors discovered during appendectomies *Other benign histopathology such as tubulovillous adenoma, benign myxoid mesenchymal lesion and endometriosis in appendix.

Pathology	N	Percentage (%)
Healthy appendix	109	7.2%
Acute Appendicitis	1,381	91.3%
Neuroendocrine tumor	3	0.2%
Adenocarcinoma	1	0.07%
Carcinoid	1	0.07%
Mucinous	10	0.7%
Others*	8	0.5%

**Table 5:** Comparisons between cancer and noncancer patient data Abbreviations: RLQ, right lower quadrant; WBC, white blood cells; NS, not significant.

Variable	Tumor (n=15), n (%)	No Tumor (n=1498), n (%)	P-value
Mean Age in Years (SD)	47.6 (21.5)	27.7 (12.0)	< .001
Male Patients	7 (46.7%)	951 (63.5%)	NS
Female Patients	8 (53.3%)	547 (36.5%)	NS
RLQ Tenderness	11 (73.3%)	1447 (96.6%)	< .001
Fever (>37.3 °C)	4 (26.6%)	224 (15.0%)	NS
Rebound Tenderness	7 (46.7%)	1106 (78.0%)	0.018
Anorexia	7 (46.7%)	851 (56.8%)	NS
Nausea and vomiting	9 (60.0%)	1169 (78.0%)	NS
Migration of pain to RLQ	2 (13.3%)	863 (57.6%)	< .001
WBC >10,000 cells/µL	7 (46.7%)	1,093 (27.0%)	0.023
Neutrophils > 75%	10 (66.7%)	887 (59.2%)	NS
Laparoscopic Procedure	14 (93.3%)	1472 (98.3%)	NS

**Figure 1:**
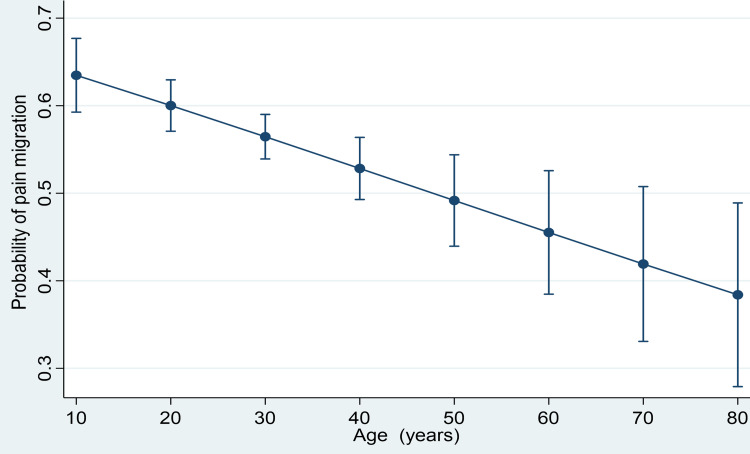
Prediction of probability of pain migration with age

Figure 1 shows the prediction of the probability of pain migration with age. Migration of pain was significantly inversely correlated with age; as age increased, the migration of pain was reported less (odds ratio, 0.99, 95 confidence interval, 0.98 to 0.99, p=0.001).

## Discussion

We analyzed 1,513 patients with appendicitis, of whom only 15 (1.0%) had appendiceal tumors. This suggests that appendiceal masses are rare in appendicitis patients, as also reported by Teixeira et al., who found that 1% of 13,244 acute appendicitis patients had appendiceal tumors [9]. They also found that the incidence of appendiceal tumor was associated with inflamed appendicitis. 

We measured all patients’ Alvarado score, which indicates likelihood of acute appendicitis and if there is a relationship between the score and malignancy. Less migration of pain was observed in patients with malignancy and higher WBC count. According to Ma et al., the level of neutrophils and leukocytes and the modified Alvarado scores are positively correlated with appendiceal neoplasms (all p<0.05) [10].

Loftus et al. conducted a retrospective analysis of pathological sample data in a tertiary medical center to determine the prevalence of occult appendiceal neoplasms and identify high-risk variables related to this pathology. Their incidence was similar to that found in our study. They found 31 appendiceal neoplasms in total (1.7%), 14 (1.0%) in cases of acute appendicitis, two (4.9%) in cases of chronic appendicitis, and 15 (3.6%) in cases when an incidental appendectomy was done (p<.001) [11]. They also found that patients with appendiceal neoplasms were generally older than those without (mean age, 53 years), which is similar to finding where neoplasms were found in older patients. 

Brunner et al. conducted a similar retrospective study from 2010 to 2016 on 1,033 patients [12]. Twenty-nine of their study participants had appendiceal neoplasm, and they were older than those without (mean age, 57 years), which is similar to the age-related findings in our study.

In our study, most patients with appendicitis (n=1,100; 72.7%) had increased WBC counts, and seven patients of them had appendiceal neoplasms. Therefore, while WBC count could be a predictor of pathological appendix, the association might not strongly predict a cancerous mass. Withers et al. observed higher WBC count in appendicitis patients and claimed that increased WBC count was associated with a higher probability of complex appendicitis [13].

In our study, most patients (95.4%) had no complications after surgery, which aligns with the results reported by Wu et al., who found that a majority (85%) of their patients had no complications after similar surgery [14]. 

Bucher et al. conducted a 10-year study of patients with appendiceal tumors treated by laparoscopy or laparotomy [15]. They found 20 malignant masses (mean age, 69 years) and 23 carcinoid lesions (mean age, 36 years; p 0.01 ). Acute appendicitis was observed in 35% of other malignancies and 70% of carcinoid patients (p<0.05). They used laparoscopic surgery to treat eight carcinoid masses and laparotomy to treat 15 masses. They used laparoscopic surgery to remove three malignant tumors and open surgery to remove 17 tumors. In our study, 14 patients received laparoscopic surgery, and one received open surgery. 

Our study had some limitations. First, we wanted a larger sample size with a longer period of time, but unfortunately all histopathology data before 2015 were not documented at the BestCare system. Second, the data were collected from a single institution. 

## Conclusions

We conducted this study to assess the prevalence and types of appendiceal tumors in cases of acute appendicitis and the corresponding patient demographic data. Patients with appendicitis present with fever, rebound tenderness, nausea, and high WBC count. Appendiceal masses primarily occurred in older patients who also had less migration of pain and high WBC count. However, migration of pain is inversely related to age. Physicians treating patients with acute appendicitis should bear these data in mind and consider the presence of appendiceal tumors in appropriate patients.
